# The Use of Global Rating Scales for OSCEs in Veterinary Medicine

**DOI:** 10.1371/journal.pone.0121000

**Published:** 2015-03-30

**Authors:** Emma K. Read, Catriona Bell, Susan Rhind, Kent G. Hecker

**Affiliations:** 1 Department of Veterinary Clinical and Diagnostic Sciences, University of Calgary Faculty of Veterinary Medicine, Calgary, Alberta, Canada; 2 Royal (Dick) School of Veterinary Studies, University of Edinburgh, Roslin, Midlothian, Scotland; Oregon State University, UNITED STATES

## Abstract

OSCEs (Objective Structured Clinical Examinations) are widely used in health professions to assess clinical skills competence. Raters use standardized binary checklists (CL) or multi-dimensional global rating scales (GRS) to score candidates performing specific tasks. This study assessed the reliability of CL and GRS scores in the assessment of veterinary students, and is the first study to demonstrate the reliability of GRS within veterinary medical education. Twelve raters from two different schools (6 from University of Calgary [UCVM] and 6 from Royal (Dick) School of Veterinary Studies [R(D)SVS] were asked to score 12 students (6 from each school). All raters assessed all students (video recordings) during 4 OSCE stations (bovine haltering, gowning and gloving, equine bandaging and skin suturing). Raters scored students using a CL, followed by the GRS. Novice raters (6 R(D)SVS) were assessed independently of expert raters (6 UCVM). Generalizability theory (G theory), analysis of variance (ANOVA) and t-tests were used to determine the reliability of rater scores, assess any between school differences (by student, by rater), and determine if there were differences between CL and GRS scores. There was no significant difference in rater performance with use of the CL or the GRS. Scores from the CL were significantly higher than scores from the GRS. The reliability of checklist scores were .42 and .76 for novice and expert raters respectively. The reliability of the global rating scale scores were .7 and .86 for novice and expert raters respectively. A decision study (D-study) showed that once trained using CL, GRS could be utilized to reliably score clinical skills in veterinary medicine with both novice and experienced raters.

## Introduction

Educating veterinary students to become competent, autonomous practitioners requires ongoing assessment of students’ abilities and performance using methods that provide reliable and valid scores. For professional skills globally, and veterinary clinical skills specifically, the Objective Structured Clinical Examination (OSCE) has become one of the key performance based methods of assessment. [1,2] While the OSCE was first reported over 30 years ago in human medicine [3] it has only relatively recently been adopted for use in veterinary medicine but is gaining widespread acceptance. [2,4,5] Advantages of the OSCE over previously used methods of assessment of clinical skills include standardization of the tasks performed by all students, the ability to use trained non-subject matter experts as raters, and the reliability of judgments made between raters. [6] It is well documented that for OSCE scores to be reliable and valid, the OSCE must have a series of timed stations, the exam should be blueprinted with a specified set of tasks that are performed in the presence of trained raters and that tasks, timing and raters are standardized over all students.[6]

Scoring methods for OSCEs are typically either analytic, meaning they incorporate binary checklists (CLs) that quantify small elements of performance as yes/no, or holistic, meaning they utilize global rating scales (GRS) that consider skill performance across several domains using a Likert–type scale. [7] The use of CLs is generally accepted in veterinary medical OSCEs and affords high inter-rater reliability if they are well written, revised after pilot testing and involves adequate rater training. [1,8] However, CLs may not be suitable in all situations and they may reward thoroughness without consideration of timeliness or proficiency of action. [9] Some CLs incorporate a global assessment (GA) at the end to allow raters to provide a subjective score of the student performance and this may or may not correlate well to the overall CL score. [4] More advanced clinical trainees have been shown to skip steps and more rapidly proceed to an endpoint while taking short cuts which could penalize them when being graded using a binary checklist. [9]

Global rating scales (GRS) have also been used in medicine to assess OSCE performance. [8, 10] Almost twenty years ago, GRS were first compared to CLs for scoring technical skills performance. [11, 12] GRS are reported to assess more qualitative performance values, such as overall preparation for the task or efficiency of performance, which can be useful, but does bring up concerns about the need for highly trained raters and inter-rater reliability to avoid subjectivity. [8,10] Despite these concerns, it is reported that assessors are able to discriminate performance better when not tied to reducing clinical performance into a number of individual steps (process-level observation). [13,14] Recently, GRS scores were shown to yield reliable data when compared with CL scores for assessment of more advanced learners (residents) in the field of medicine. [8, 10,15] To date, only one recent report has discussed the use of GRS for the assessment of clinical skills proficiency in veterinary students, however, these authors only addressed issues of pre and post score student satisfaction with the tool and did not demonstrate whether scores from the GRS tool were reliable or valid. [5]

To explore the use of GRS for assessment of veterinary clinical skills, student and rater performance in the context of two different veterinary programs were compared using a fully crossed design in which all students were rated on all stations, by all raters, from standardized video recordings of four different OSCE stations.

The purpose of this study was twofold: First, to assess novice and expert rater performance differences on CL and GRS scores, and second, to assess the reliability of CL and GRS scores from both types of raters. While previous studies have compared CL and GRS scores, this study adds to the literature by providing a direct comparison of novice and expert raters and demonstrates that CLs can be used to inform the use of GRS with both types of raters.

## Materials and Methods

### Institutions

The University of Calgary Faculty of Veterinary Medicine (UCVM) in Canada, was established in 2005, accepted its first cohort of students in 2008. This program has a heavy emphasis on clinical skills training with approximately 20% of each year’s curriculum devoted to formalized training beginning in first year, with regular assessment using summative OSCEs. [16] The Royal (Dick) School of Veterinary Studies (R(D)SVS) at the University of Edinburgh was established in 1823, and has clinical skills training throughout the 4 and 5-year programs that it offers. Clinical and practical skills are assessed using different summative practical examinations aligned to specific courses, often as a short series of stations, but there is no large multiple station OSCE format in place.

### Participants

Video recordings of student performances on all four OSCE stations were completed in April and May 2013. The six students from UCVM (5 females and 1 male) had recently completed their first year of the four-year veterinary program resulting in 120 hours each of dedicated clinical skills training, and 3 summative multi-station OSCEs. The six students from R(D)SVS (5 females and 1 male) had partially completed their third year of the five-year veterinary program and had 15 hours of dedicated clinical skills training (with no summative OSCE’s) plus additional practical skills training in two other courses (with associated summative assessments).

Twelve raters were identified from UCVM and R(D)SVS (6 from each school). The raters from UCVM (four females, 2 males) had 1–6 years experience rating OSCEs and were clinicians who had been trained on other occasions using the CL assessment method by 2 of the authors (EKR and KGH). The R(D)SVS raters (5 females, 1 male) had no previous experience assessing OSCEs. All ratings were completed between July and September 2013.

### Ethics Statement

The study was approved by conjoint health research ethics board at the University of Calgary, and the Medicine and Veterinary Medicine Education Research (EREC) Project ethics board at the University of Edinburgh.

Informed written consent was obtained from all study participants prior to participation in the study. The consent procedure was approved by the ethics board of each institution.

### Measures

The OSCE stations used in this study were previously developed at UCVM and included bovine haltering, skin suturing, equine bandaging, and surgeon preparation (gowning and gloving). [4] The original OSCE checklists were modified, for the purpose of this study, by EKR and CB. Each checklist was binary, comprising 8 to 40 items. A student’s CL score for a given station was the sum of the number of items that were scored as yes. Station scores were converted to mean percent scores.

**Fig 1:**
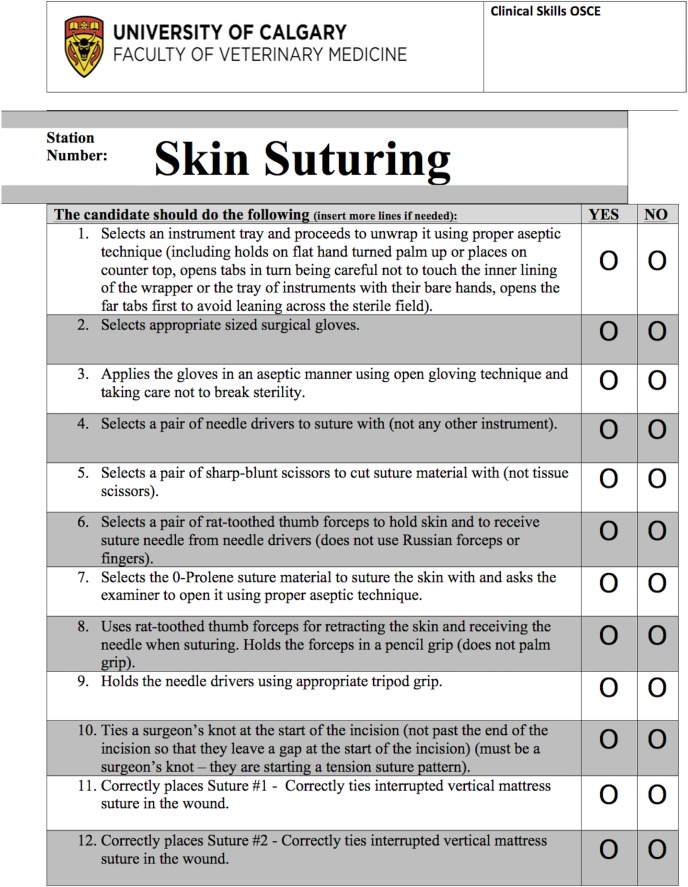
Example checklist instrument for the skin suturing station.

**Fig 2:**
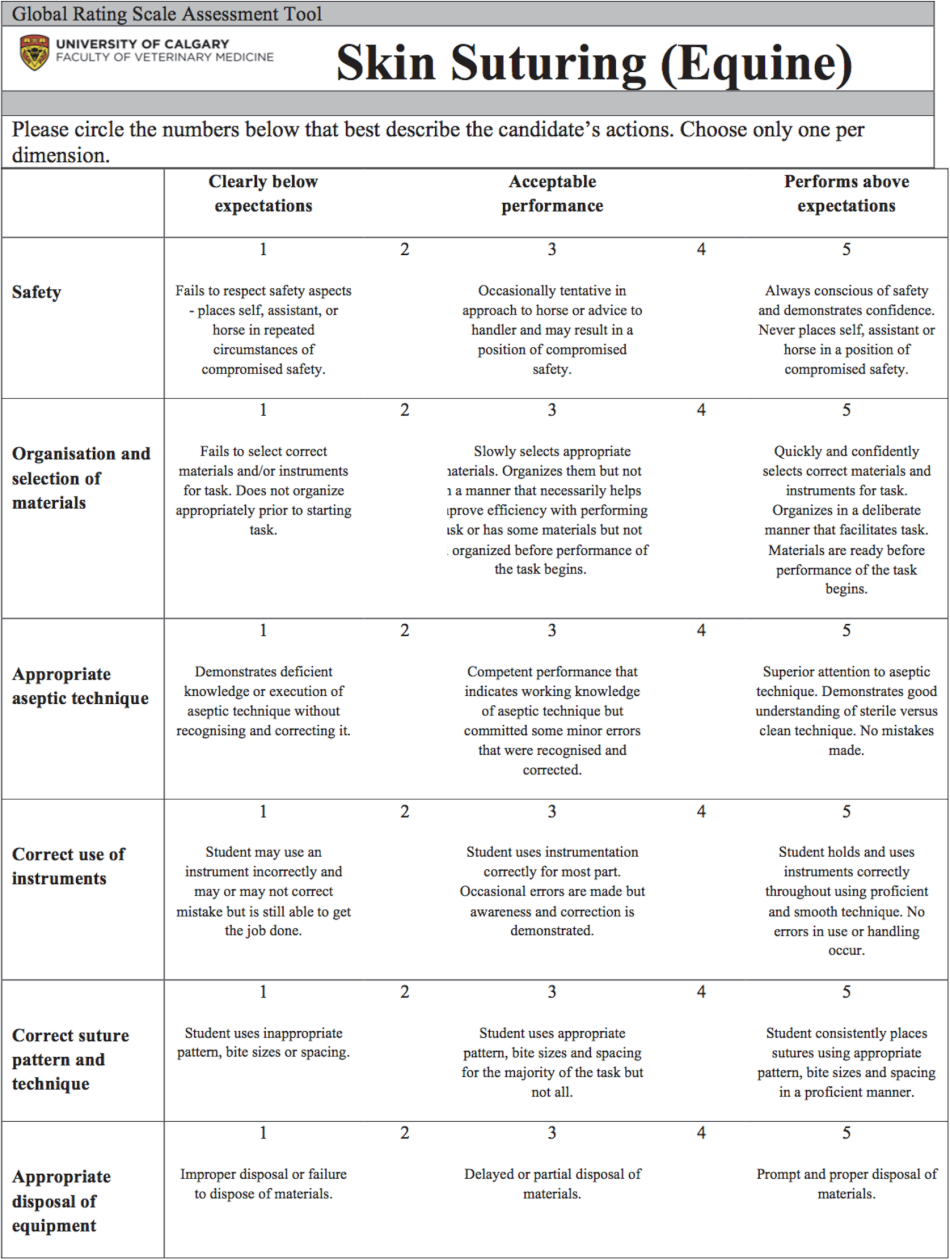
Example global rating scale instrument for the skin suturing station.

The GRS were developed using the checklists as a reference to allow grouping of similar skills into dimensions that were assessed using a 5-point scale, with points 1, 3, and 5 being anchored by explicit descriptors (1 being the lowest level of performance and 5 being the highest level of performance). Each GRS had between 6 and 8 dimensions corresponding to specific groupings of checklist items into broader categories. Other dimensions such as time and motion, that were not related directly to the checklist items, were also developed. A student’s GRS score for a given station was the sum of each 5 point item over the entire tool. Station scores were converted to mean percent scores. See Fig. 1 for a CL example and Fig. 2 for a GRS example.

### Evaluations

Each student was video recorded performing each of the 4 stations in the same order. Raters were not trained in the use of the assessment tools (beyond their prior experience) but were given a brief explanation by the authors (EKR, CB) as to the purpose of each tool prior to their use if requested. All raters independently rated all students from all schools. Each rater was asked to perform the CL rating first and then the GRS second for each student.

### Analyses

Generalizability theory was used to calculate the reliability of the OSCE scores and to perform a decision (D) study. We ran two generalizability analyses, one for the UCVM (expert) rater data and one for the R(D)SVS (novice) rater data. The generalizability (G) studies were fully crossed designs with the following facets, participants (students) (12), stations (4), and raters (6). A decision (D) study was then run to identify the optimal number of stations given one or two raters per station. A two-way Analysis of Variance (ANOVA) was used to assess rater and student school differences in CL and GRS scores. Dependent *t* tests were used to assess differences in CL and GRS scores by the same rater.

## Results

Rater CL and GRS scores (mean percent and standard deviation) for each of the OSCE stations and all stations combined are presented in Table 1. The two–way ANOVAs revealed a significant difference in student performance by school in both the CL (*F*_(1,140)_ = 78.53, p <. 001) and GRS scores (*F*_(1,140)_ = 105.84, p <. 001) with the R(D)SVS students performing significantly lower than UCVM students. There was no difference between rater scores from the two schools and there was no interaction effect between rater school and school that the students attended. There were significant differences in rater scores using CL versus GRS regardless of rater school. Specifically, total CL scores were significantly higher than total GRS scores (UCVM *t*(71) = 9.17, p<0.001; RDSVS *t*(71) = 15.11, p<0.001).

**Table 1:** Mean percent scores (and standard deviations) from UCVM and R(D)SVS raters for OSCE Checklist and Global rating scores.

	****Checklist****		****Global Rating Score****	
	****CL—UCVM****	****CL—R(D)SVS****	****GRS—UCVM****	****GRS—R(D)SVS****
**Station1**	90.59 (8.16)	91.60 (8.35)	68.89 (18.99)	71.11(14.90)
**Station 2**	84.03(16.28)	77.60 (17.16)	70.79(14.91)	68.06(16.21)
**Station 3**	72.98 (16.64)	73.48 (17.91	72.61(16.51)	72.11(17.71)
**Station 4**	60.46 (16.15)	66.76(14.82)	58.06(18.39)	56.30(16.67)
**Total Score**	77.02 (10.26)	77.36 (8.77)	67.59 (13.12)	66.89 (10.86)

CL—checklist; GRS—global rating scale; UCVM—UCVM raters; RDSVS—R(D)SVS raters

The reliability of the CL and GRS scores were. 76 and. 86 respectively for UCVM raters suggesting that scores for students across raters and stations were generally consistent (see Table 2). The reliability of the CL and GRS from R(D)SVS raters were. 42 and. 70 suggesting scores weren’t as consistent across raters and stations for CL scores as they were for the GRS scores. The amount of variance for students (participants; p) was higher in the GRS scores compared to the CL scores for both groups of raters. Interestingly the amount of variance accounted for by raters was relatively low (3.76%, 3.87%, 4.68%) except for the GRS-UCVM value at 15.61%. Variability due to rater, especially when variance due to rater has been higher than variance due to student/participant, has often been identified as a source of error and has caused much discussion regarding rater training and selection for OSCEs and clinical observations [17,18]. The station facet (s) accounted for considerably more variance in checklist scores from both groups of raters (42.22% UCVM, 28.65% R(D)SVS) than in GRS scores (9.57% UCVM, 13.03% R(D)SVS). The participant (student) by station (p|s) facet for the R(D)SVS raters was 24.45% and lower (9.29%) for the UCVM raters for CL scores and there was a similar trend in the GRS scores (21.06% R(D)SVS and 10.36% UCVM respectively). While the trend in the GRS was for a higher G-coefficient and higher variance due to participant, unsystematic error and varying scores of students across raters and stations accounted for 31.95% and 37.49% of the variation respectively in the UCVM and R(D)SVS scores.

**Table 2:** Generalizability (G) study (for participant (student) (12), rater (6) and station (4)) and Decision (D) study with reliability coefficient (G-coef) calculated using the variance components.

	**CL—UCVM**		**CL—R(D)SVS**		**GRS—UCVM**		**GRS—R(D)SVS**	
	**σ** ^2^	**%**	**σ** ^2^	**%**	**σ** ^2^	**%**	**σ** ^2^	**%**
**p**	47.23	11.94	17.79	5.23	88.11	24.92	54.49	16.77
**r**	15.31	3.87	15.91	4.68	55.20	15.61	12.23	3.76
**s**	167.02	42.22	97.44	28.65	33.83	9.57	42.33	13.03
**p|r**	11.05	2.79	0.00	0.00	1.49	0.42	6.64	2.04
**p|s**	36.75	9.29	83.16	24.45	36.62	10.36	68.42	21.06
**r|s**	18.87	4.77	26.14	7.69	25.37	7.17	19.00	5.85
**p|r|s, error**	99.36	25.12	99.65	29.30	112.95	31.95	121.80	37.49
**G-coef**	0.76		0.42		0.86		0.70	
**D- study values**								
**raters**	**stations**	**CL—UCVM**	**CL—R(D)SVS**	**GRS—UCVM**	**GRS—R(D)SVS**			
2	4	.64	.35	.79	.60			
1	4	.51	.28	.69	.50			
2	5	.67	.40	.82	.65			
1	5	.55	.33	. 74	.55			
2	6	.70	.45	.84	.69			
1	6	.58	.37	.77	.59			
2	7	.73	.48	.86	.71			
1	7	.61	.41	.79	.62			
2	8	.74	.52	.88	.74			
1	8	.63	.44	.81	.64			
2	9	.76	.55	.89	.75			
1	9	.64	.47	.83	.66			
2	10	.77	.57	.90	.77			
1	10	.66	.49	.84	.68			
2	11	.78	.60	.91	.78			
1	11	.67	.52	.85	.69			
2	12	.79	.62	.91	.79			
1	12	.68	.54	.86	.79			

CL—checklist; GRS—global rating scale; UCVM—UCVM raters; R(D)SVS—R(D)SVS raters p- participant (student); r—rater; s- station; **σ** ^2−^variance; %—percentage.

The D-study determined that more than 12 stations (using the benchmark of a reliability coefficient of 0.7) are required for OSCE scores to be reliable when using one rater per station for both UCVM and R(D)SVS using CLs. For GRS the D study determined that at least 5 stations are required for UCVM raters but you would need at least 12 for the R(D)SVS raters for their scores to achieve an acceptable level of reliability when using one rater per station.

## Discussion

The findings from this study were the following. First, there was no difference in rater scores of student performance between schools using CL and GRS; second, scores from CLs were significantly higher than scores from GRS; and third, scores from GRS regardless of rater type, demonstrated greater reliability than CL, where the use of CL preceded the use of GRS.

CL scores were greater than GRS scores, which is not surprising given that the GRS allow raters greater opportunity to assess other dimensions not represented in CL such as time, efficiency, motion and safety. This has also been identified in previous work. [10, 19] Two different students can perform the same task and score “yes” for the same CL items but have differing performance. A student may quickly and efficiently perform the items ending up with a similar score to a student who slowly and repeatedly performs the items until they eventually obtain a yes for each one. This implies that, at least for some tasks, it may not be possible to sufficiently discriminate between candidates using a simple binary CL. [10, 15,19] GRS provides the opportunity to score additional dimensions that separate a superior performance from an average or poor one, and provide the opportunity for more qualitative feedback. [19] It is also important to consider that GRS may be subject to more inherent rater biases of student performance, these biases could range from personal preference of particular technique/method to be used in a station to age/appearance or gender biases. [10, 19] However, in this study we did not explore these biases with the participating raters. Conversely, CLs focus raters on the parts of the task because they are based on directly observable behaviors, e.g. the student either performed the items or did not. The findings from this study are not surprising and similar to those reported elsewhere. [10]

Scores from raters who had not been trained (R(D)SVS) demonstrated lower reliability because they had not been exposed to rater training, were not familiar with the assessment tool or testing process in advance, and did not have an opportunity to practice scoring, thus supporting the widely recognized importance of rater training prior to the use of any new assessment tool (including CLs). [1,6,9] However, while the reliability coefficients were lower, the same general trend existed between novice and expert raters with no significant differences in student performance by rater type.

Scores from the GRS were more reliable for UCVM and R(D)SVS raters. This might be due to (1) the way in which the scale was constructed and utilized, perhaps making it easier to use than the CL, (2) the possibility that raters developed familiarity with the items while using the CL that in turn made the GRS easier to use, or (3) the possibility that raters developed familiarity with student performances while using the CL that improved the precision of their judgments while using the GRS. Regardless, the reliability was found to be adequate for both the expert and the novice raters. Furthermore, the D studies performed demonstrated that 5 stations with one expert rater per station had a reliability of. 74. For novice raters the D study showed that 12 stations with one rater per station would be required to achieve a similar reliability with GRS. Conversely, greater than 12 stations would be required to achieve a reliability of greater than. 7 when using CL alone no matter whether expert (G = .68) or novice (G = .54) as a rater.

It is important to recognize that there are limitations to our study. First of all, the order of tool use was not randomized and the CL was always scored before the GRS. CL’s were used first because this follows the logical order associated with development of these tools. Typically, the CL is created using a cognitive task analysis or some other rigorous development approach, and then the GRS is created, by grouping these individual items into dimensions. Missing factors are then included such as safety, attention to sterile technique, time and motion. Our results may indicate that the CL does inform the consistency of the measurement of the GRS. Interestingly, Cunnington et al. compared CL and GRS scores from experienced raters, whereby two independent raters assessed the same student concurrently with different tools. They found that the tools performed similarly, with GRS being at least as reliable as CL scoring. [19] Their study did not evaluate the effect of rater experience or training as was carried out here. The results from their study and our own indicates that scores from the GRS are at least as reliable as CL scores; [19]

Another limitation may be the perceived smaller sample size of both raters and students. Due to the fully crossed study design, raters reviewed 96 recorded OSCE interactions (12 students x 4 stations x 2 tools). This design allowed for all raters to rate all students across all stations yielding variance components for all facets, as opposed to typical OSCE data where raters are nested within station. In this study, we were able to identify the variance due to type of rater (novice, expert), participant (student), and station thus providing a clear understanding of where the error variance occurs.

The raters were asked to assess the students following observation of video recordings. One could criticize that raters might have been unable to see all of the student’s performance depending on camera angles or image quality compared to a live experience where the examiner is free to move around to shift perspective. Based on a previous study documenting that video recording is a valuable assessment tool for OSCE grading with both CL and GRS, we felt justified to use this method to compare two different school populations of raters and students. [20]

## Conclusions

This study has demonstrated for the first time within veterinary medical education that scores from GRS demonstrate an acceptable level of reliability and may allow for better discrimination than CL between students of varying qualities by both novice and experienced raters. This determination of a good versus average performance becomes more critical as a student advances in their training. The use of CLs may be helpful to inform raters and allow training for GRS use, but this study also confirmed that the use of CL without training of novice raters did not result in a reliable assessment methodology.

## Supporting Information

S1 Data G Study global score percent and RS (compressed SAV file). (ZIP)

S2 Data G Study checklist (compressed SAV file). (ZIP)
